# Coronary Artery Bypass Grafting Plus Mitral Valve Plasty May Not Provide More Advantage in Patients with Coronary Heart Disease and Moderate Ischemic Mitral Regurgitation: An Inverse Probability of Treatment Weighting Retrospective Cohort Study

**DOI:** 10.21470/1678-9741-2023-0254

**Published:** 2024-11-18

**Authors:** Kui Zhang, Wei Fu, Kaiwen Liu, Junhang Jia, Yueli Wang, Xiaoyan Gu, Han Zhang, Taoshuai Liu, Yue Song, Jian Cao, Jubing Zheng, Ran Dong

**Affiliations:** 1 Department of Cardiac Surgery, Beijing Anzhen Hospital, Capital Medical University, Beijing, People’s Republic of China; 2 Department of Echocardiography, Beijing Anzhen Hospital, Capital Medical University, Beijing, People’s Republic of China

**Keywords:** Coronary Heart Bypass, Coronary Disease, Mammary Arteries, Mitral Valve Insufficiency, Treatment Outcome

## Abstract

**Objective:**

To compare the efficacy of isolated off-pump coronary artery bypass grafting (OPCABG) and of coronary artery bypass grafting (CABG) plus mitral valve plasty (MVP) in treating coronary heart disease with moderate ischemic mitral regurgitation to find a better surgical method.

**Methods:**

Clinical data of 822 patients diagnosed with coronary heart disease and moderate ischemic mitral regurgitation were analyzed retrospectively. Patients were divided into the OPCABG and CABG+MVP groups according to surgical methods. Baseline data of both groups were corrected, and clinical efficacy of the two surgical methods was analyzed and compared using the propensity score inverse probability of treatment weighting (IPTW) method.

**Results:**

There were no significant differences in the use of mammary artery grafts, number of grafts, and blood product consumption between the two groups (P>0.05) after IPTW. However, the CABG+MVP group had a significantly longer operation time than the OPCABG group (4.13 ± 0.85 hours vs. 5.65 ± 1.02 hours, P<0.001). No statistically significant differences in postoperative major adverse cardiac and cerebrovascular events were observed between the two groups. However, the intra-aortic balloon pump rate was higher in the CABG+MVP group than in the OPCABG group (12.3% vs. 25.0%, P=0.012). Although CABG+MVP can improve ischemic mitral regurgitation significantly (95.4% vs. 81.2%, P<0.001), there were no significant differences in the cumulative survival rate and the incidence of major adverse cardiac and cerebrovascular events between the groups (P>0.05) after IPTW.

**Conclusion:**

CABG+MVP may not provide more advantage in patients with coronary heart disease and moderate ischemic mitral regurgitation.

## INTRODUCTION

**Table:** 

Abbreviations, Acronyms & Symbols				
AKI	= Acute kidney injury		IPTW	= Inverse probability of treatment weighting
AMI	= Acute myocardial infarction		IQR	= Interquartile range
BMI	= Body mass index		LCOS	= Low cardiac output syndrome
BNP	= Brain natriuretic peptide		LIMA	= Left internal mammary artery
BSA	= Body surface area		LVEDD	= Left ventricular end-diastolic diameter
CABG	= Coronary artery bypass grafting		LVESD	= Left ventricular end-systolic diameter
CHD	= Coronary heart disease		MACCE	= Major adverse cardiac and cerebrovascular events
CNSD	= Central nervous system disease		MI	= Myocardial infarction
COPD	= Chronic obstructive pulmonary disease		MR	= Mitral regurgitation
CPB	= Cardiopulmonary bypass		MVP	= Mitral valve plasty
CrCl	= Creatinine clearance		OPCABG	= Off-pump coronary artery bypass grafting
CRRT	= Continuous renal replacement therapy		PCI	= Percutaneous coronary intervention
CTSN	= Cardiothoracic Surgical Trials Network		PVD	= Peripheral vascular disease
EF	= Ejection fraction		SD	= Standard deviation
GFR	= Glomerular filtration rate		SMD	= Standardized mean difference
IABP	= Intra-aortic balloon pump		TNI	=Troponin I
ICU	= Intensive care unit		TR	= Tricuspid regurgitation
IMR	= Ischemic mitral regurgitation			

Ischemic mitral regurgitation (IMR) is a common complication of coronary heart disease (CHD)^[1]^. Surgery, mainly including coronary artery bypass grafting (CABG) and combined mitral valve plasty (MVP), is an effective treatment for patients with CHD with IMR^[2]^. However, for those with moderate IMR, there has been controversy as to whether MVP should be performed during CABG^[3,4]^. The clinical data of surgical treatment of CHD with moderate IMR complication in the recent 10 years from our hospital were retrospectively analyzed to compare the efficacy of off-pump CABG (OPCABG) and CABG+MVP in treating CHD with IMR complication.

## METHODS

### Study Population

A retrospective analysis was performed on 998 patients diagnosed with CHD combined with moderate IMR who underwent surgery between January 2012 and December 2021 at our hospital. The inclusion criteria included: ① patients diagnosed with CHD by coronary angiography and needing CABG; ② patients with moderate IMR (regurgitation area 4-8 cm^2^) diagnosed by resting transthoracic echocardiography; and ③ patients with complete clinical data. The exclusion criteria included: ① organic mitral regurgitation (MR) (rheumatism, degeneration, and infective endocarditis, among others); ② MR caused by rupture of mitral chordae tendinae or rupture of papillary muscle in acute myocardial infarction (MI); ③ CABG combined with other cardiac surgery (such as aortic valve, congenital heart disease, great vascular disease, and ventricular aneurysm resection); (4) patients with preoperative atrial fibrillation; (5) patients with preoperative ventricular aneurysm; (6) patients with preoperative malignant tumors; and (7) on-pump CABG.

**Fig. 1:**
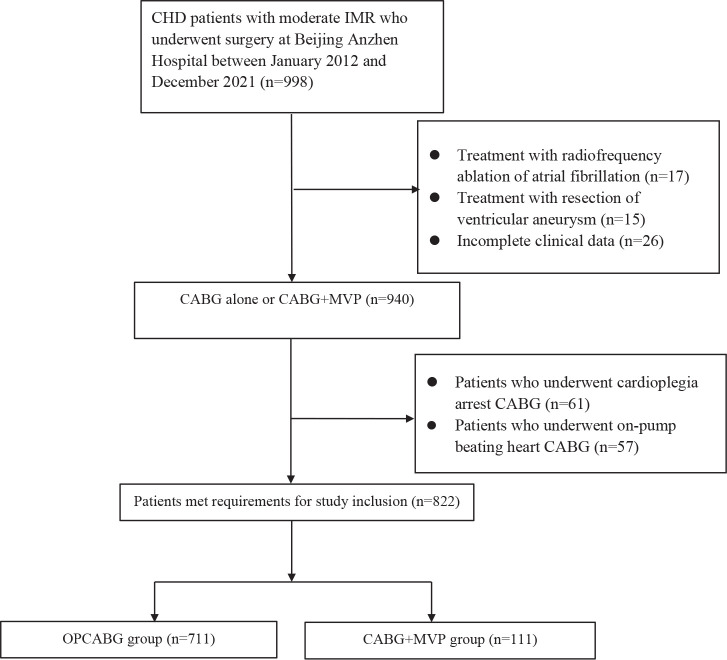
Flow chart for the selection of study participants. CABG=coronary artery bypass grafting; CHD=coronary artery disease; IMR=ischemic mitral regurgitation; MVP=mitral valve plasty; OPCABG=off-pump coronary artery bypass grafting.

Of the 998 patients, 17 underwent radiofrequency ablation of atrial fibrillation, 15 underwent ventricular aneurysm resection, 26 had incomplete clinical data, 61 underwent cardioplegia arrest CABG, and 57 patients underwent on-pump beating heart CABG and thus were excluded. Therefore, the remaining 822 patients were included in this study and were divided into the OPCABG group (711 cases) and the CABG+MVP group (111 cases) according to the surgical method (Figure 1).

### Study Protocol

Patients were divided into the OPCABG group (711 cases) and the CABG+MVP group (111 cases) based on the surgery they underwent. The baseline data of the two groups were balanced, and the clinical efficacy of the two surgical methods was compared using the inverse probability treatment weighting (IPTW) method to reduce the impact of treatment selection bias and potential differences on the outcome. This study met the requirements of the Declaration of Helsinki. The data use was approved by the Ethics Committee of Beijing Anzhen Hospital, Capital Medical University (approval number: 2016024). The requirement to obtain informed consent from patients was waived since this was a retrospective observational cohort study.

### Surgical Technique

All the operations were performed using tracheal intubation under general anaesthesia. The patients were placed on the operating room table in a supine position, and the median sternal incision was made. OPCABG or CABG+MVP was performed by experienced surgeons. The internal mammary artery was obtained by ossification or pedicle technique, and the first choice was the left internal mammary artery graft to the left anterior descending branch. The great saphenous vein was obtained by open technique. Then, the circumflex branch and the right coronary artery were anastomosed. The quality of graft anastomosis was evaluated using transient time flow measurement. The mitral valve was detected via the atrial groove or left atrial-atrial septal approach. The distance between the anterior and posterior interfaces was measured using an annulus detector. A 2 mm semi-hard and semi-soft ring was selected for MVP. Routine antiplatelet and CHD drugs were administered to all patients after surgery^[5]^.

### Observation Indicators and Follow-up

Intraoperative and postoperative data, postoperative complications, and major adverse cardiac and cerebrovascular events (MACCE) during follow-up were observed in the two groups. Intraoperative data included the number of grafts, operation time, and blood product consumption, among others. Postoperative data included intensive care unit (ICU) length of stay, length of time on a ventilator, and postoperative echocardiography results (collected seven days after surgery), among others. Postoperative complications included perioperative death, MI, heart failure, cerebrovascular events, secondary thoracotomy, secondary tracheal intubation, acute kidney injury (AKI), and infections. Meanwhile, the use of an intra-aortic balloon pump (IABP) and continuous renal replacement therapy (CRRT) were also recorded. Follow-up data were obtained by the outpatient department, telephone call, or WeChat. MACCE events included all-cause death, MI, heart failure, cerebral infarction, re-revascularization, and rehospitalization due to heart disease. All data were collected from our online database by trained staff.

### Statistical Analysis

Normally distributed data were expressed as mean ± standard deviation. Differences between groups of normally distributed data were evaluated by the *t*-test. Non-normally distributed data were expressed with median and interquartile distance (median [P25, P25]) and were analyzed by the Mann-Whitney U test. Count data were expressed using the chi-square or Fisher’s exact test as frequency (rate). The probability of receiving CABG+MVP (*i.e.*, propensity score) for each patient was calculated using binary logistic regression analysis based on these 21 variables: sex, age, body mass index, body surface area (BSA), history of hypertension, diabetes, history of hyperlipidemia, history of percutaneous coronary intervention, history of MI in the last three months, smoking, history of central nervous system disease, history of chronic obstructive pulmonary disease, history of peripheral vascular disease, troponin I, creatinine clearance rate, glomerular filtration rate, left ventricular ejection fraction, left ventricular end-diastolic diameter (LVEDD), left ventricular end-systolic diameter (LVESD), MR area, and tricuspid regurgitation grade. IPTW for the propensity score was calculated using the normalization method to make the distribution of propensity scores consistent between the two groups. We evaluated the standardized mean difference (SMD) before and after IPTW to measure whether the covariates were balanced. SMD < 0.10 indicated that the comparison between the two groups was balanced. The survival curve was drawn using the Kaplan-Meier algorithm, and whether there were differences in the survival curve between the two groups was determined using the Log-Rank test. All tests were bilateral, and *P*<0.05 was considered statistically significant. In this study, data analysis was performed using R software, version 4.1.2.

## RESULTS

### Baseline Data

A total of 822 CHD patients with moderate IMR that met the inclusion criteria were enrolled in this study. The baseline data of the two groups are shown in Table 1. Before IPTW, the CABG+MVP group had fewer females (30.8% *vs.* 18.0%, *P*=0.008) and younger individuals (63.43 ± 8.68 years *vs.* 61.34 ± 10.09 years, *P*=0.022) than the OPCABG group. The BSA was larger (1.74 ± 0.18 m^2^ *vs.* 1.80 ± 0.16 m^2^, *P*=0.001), and the LVEDD, LVESD, and MR area were larger (52.95 ± 6.81 mm *vs.* 55.47 ± 7.22 mm, *P* < 0.001; 37.73 ± 7.95 mm *vs.* 41.22 ± 8.1 mm, *P*<0.001; 5.63 ± 0.96 cm^2^ *vs.* 6.11 ± 1.10 cm^2^, respectively, *P*<0.001) in the CABG+MVP group than in the OPCABG group. After IPTW, the SMD of baseline data in both groups was < 0.1.

**Table 1:** Comparison of baseline data between the two groups before and after IPTW.

Variables	Original cohort (crude)				IPTW		
	OPCABG	CABG+MVP	*P*-value	SMD	OPCABG	CABG+MVP	SMD
	(n=711)	(n=111)			(n=1697)	(n=1721.5)	
Clinical variables							
Female (%)	219 (30.8)	20 (18.0)	0.008	0.301	453.4 (26.7)	452.8 (26.3)	0.009
Age, years, mean (SD)	63.43 (8.68)	61.34 (10.09)	0.022	0.222	62.95 (8.88)	63.45 (8.77)	0.058
BMI, mean (SD)	24.79 (2.99)	25.34 (3.18)	0.074	0.178	24.85 (2.99)	24.84 (3.16)	0.002
BSA, mean (SD)	1.74 (0.18)	1.80 (0.16)	0.001	0.362	1.75 (0.17)	1.76 (0.16)	0.036
Smoking (%)	304 (42.8)	58 (52.3)	0.076	0.191	768.4 (45.3)	758.5 (44.1)	0.025
TnI, median (IQR)	0.05 (0.01, 0.29)	0.03 (0.01, 0.26)	0.471	0.071	0.05 (0.01, 0.37)	0.04 (0.01, 1.23)	0.051
CrCl, median (IQR)	79.98 (64.60, 97.33)	84.92 (68.56, 103.83)	0.110	0.165	81.03 (65.34, 98.30)	80.72 (60.86, 102.70)	0.033
GFR, median (IQR)	66.92 (56.31, 79.89)	69.33 (55.93, 82.01)	0.534	0.066	67.06 (56.58, 79.91)	68.40 (53.77, 78.28)	0.057
Comorbidities							
Hypertension (%)	417 (58.6)	60 (54.1)	0.418	0.093	940.5 (55.4)	940.2 (54.6)	0.016
Diabetes (%)	252 (35.4)	47 (42.3)	0.194	0.142	666.5 (39.3)	656.1 (38.1)	0.024
Hyperlipidemia (%)	122 (17.2)	24 (21.6)	0.312	0.113	361.8 (21.3)	353.0 (20.5)	0.020
PCI history (%)	94 (13.2)	22 (19.8)	0.087	0.178	236.6 (13.9)	284.0 (16.5)	0.071
MI history (%)	202 (28.4)	23 (20.7)	0.115	0.179	550.3 (32.4)	575.4 (33.4)	0.021
COPD (%)	15 (2.1)	3 (2.7)	0.961	0.039	22.9 (1.3)	25.5 (1.5)	0.011
CNSD (%)	62 (8.7)	16 (14.4)	0.084	0.179	167.0 (9.8)	149.5 (8.7)	0.040
PVD (%)	248 (34.9)	39 (35.1)	1.000	0.005	652.2 (38.4)	666.3 (38.7)	0.006
Echocardiographic data							
EF, mean (SD)	52.79 (10.15)	51.53 (9.66)	0.223	0.127	51.61 (10.25)	51.36 (10.71)	0.024
LVEDD, mean (SD)	52.95 (6.81)	55.47 (7.22)	< 0.001	0.359	53.24 (6.79)	53.11 (6.66)	0.020
LVESD, mean (SD)	37.73 (7.95)	41.22 (8.10)	< 0.001	0.435	38.32 (7.99)	38.64 (7.02)	0.043
MR area, mean (SD)	5.63 (0.96)	6.11 (1.10)	< 0.001	0.470	5.69 (0.98)	5.64 (1.06)	0.044
TR grading (%)			< 0.001	0.403			0.100
No	281 (39.5)	42 (37.8)			650.7 (38.3)	676.2 (39.3)	
Mild	381 (53.6)	52 (46.8)			924.5 (54.5)	913.2 (53.0)	
Moderate	49 (6.9)	9 (8.1)			121.8 (7.2)	124.2 (7.2)	
Severe	0 (0.0)	8 (7.2)			0.0 (0.0)	8.0 (0.5)	

BMI=body mass index; BSA=body surface area; TnI=Troponin I; CABG=coronary artery bypass grafting; CNSD=central nervous system disease; COPD=chronic obstructive pulmonary disease; CrCl=creatinine clearance; EF=ejection fraction; GFR=glomerular filtration rate; IPTW=inverse probability of treatment weighting; IQR=interquartile range; LVEDD=left ventricular end-diastolic diameter; LVESD=left ventricular end-systolic diameter; MI=myocardial infarction; MR=mitral regurgitation; MVP=mitral valve plasty; OPCABG=off-pump coronary artery bypass grafting; PCI=percutaneous coronary intervention; PVD=peripheral vascular disease; SD=standard deviation; SMD=standardized mean difference; TNI=; TR=tricuspid regurgitation

### Intraoperative and Postoperative Data

Surgical data of both groups are shown in Table 2. There were no significant differences in the utilization rate of the left internal mammary artery, the number of grafts, and blood product consumption between the groups (*P*>0.05) after IPTW. Compared with the OPCABG group, the operation time of CABG+MVP group was longer (4.13 ± 0.85 hours *vs.* 5.65 ± 1.02 hours, *P*<0.001).

**Table 2:** Comparison of operation-relevant data between the OPCABG and CABG+MVP groups after IPTW.

Surgery-related indicators of patients with IPTW			
Variables	OPCABG	CABG+MVP	*P*-value
	(n=1697)	(n=1721.5)	
LIMA (%)	1059.6 (62.4)	962.3 (55.9)	0.308
Number of grafts (%)	3.21 (0.89)	3.16 (0.72)	0.516
Operation time, mean (SD)	4.13 (0.85)	5.65 (1.02)	< 0.001
CPB time, mean (SD)	-	158.65 (44.95)	-
Cross-clamping time, mean (SD)	-	97.85 (41.03)	-
Erythrocyte (U), median (IQR)	4.00 (2.00, 6.00)	4.00 (2.00, 6.32)	0.241
Plasma (ml), median (IQR)	400 (400, 600)	400 (200, 400)	0.536
Platelet (U), median (IQR)	1.00 (1.00, 3.28)	1.00 (1.00, 2.00)	0.119

CABG=coronary artery bypass grafting; CPB=cardiopulmonary bypass; IPTW=inverse probability of treatment weighting; IQR=interquartile range; LIMA=left internal mammary artery; MVP=mitral valve plasty; OPCABG=off-pump coronary artery bypass grafting; SD=standard deviation

### Postoperative Data and Complications

The postoperative data and complications of patients are shown in Table 3. The MR significantly improved in the CABG+MVP group (81.2% *vs.* 95.4%, *P*<0.001) compared to the OPCABG group after IPTW. The postoperative ICU stay, length of time on a ventilator, and troponin I were significantly higher in the CABG+MVP group than in the OPCABG group. However, no statistical difference (*P*>0.05) was observed between the two groups regarding postoperative ICU stay, length of time on a ventilator, and troponin I. No significant differences between the two groups were observed regarding secondary thoracotomy, secondary tracheal intubation, postoperative low cardiac output syndrome (LCOS), postoperative infection, postoperative AKI, postoperative nervous system injury, postoperative acute MI, postoperative CRRT utilization rate, and perioperative death. However, the CABG+MVP group had a higher rate of IABP use (12.3% *vs.* 25.0%, *P*=0.012) than the OPCABG group (Table 3).

**Table 3:** Comparison of postoperative data and complications between the two groups after IPTW.

Variables	OPCABG	CABG+MVP	*P*-value
	(n=1697)	(n=1721.5)	
ICU time (h), median (IQR)	24.50 (20.00, 49.00)	44.39 (22.00, 70.84)	0.066
Ventilator time (h), median (IQR)	21.50 (16.00, 40.00)	30.38 (19.00, 65.10)	0.238
Postoperative TnI, median (IQR)	0.26 (0.07, 0.88)	0.92 (0.34, 2.59)	0.113
Postoperative BNP, median IQR)	510.02 (296.67, 1004.57)	480.79 (294.66, 879.83)	0.725
Postoperative creatinine, median IQR)	73.40 (59.80, 88.23)	72.97 (59.08, 87.81)	0.417
Postoperative EF, mean (SD)	51.07 (9.84)	51.46 (9.91)	0.788
Postoperative LVEDD, mean (SD)	49.64 (6.63)	48.60 (5.88)	0.148
Postoperative LVESD, mean (SD)	35.69 (5.18)	36.25 (4.48)	0.398
Postoperative MR improvement (%)	1174.4 (81.2)	1527.8 (95.4)	< 0.001
Postoperative TR (%)			0.153
No	731.0 (50.7)	864.8 (55.6)	
Mild	605.5 (42.0)	655.4 (42.2)	
Moderate	94.8 (6.6)	33.9 (2.2)	
Severe	9.7 (0.7)	0.0 (0.0)	
Secondary thoracotomy (%)	36.8 (2.2)	9.2 (0.5)	0.142
Noninvasive ventilator used (%)	40.8 (2.4)	49.4 (2.9)	0.738
Secondary endotracheal intubation (%)	52.7 (3.1)	32.4 (1.9)	0.391
Postoperative LCOS (%)	225.3 (13.3)	443.3 (25.8)	0.016
Postoperative AKI (%)	69.1 (4.1)	139.4 (8.1)	0.300
Sternal dehiscence (%)	0.0 (0.0)	19.7 (1.1)	0.322
Infection (%)	149.9 (8.8)	178.4 (10.4)	0.650
Neurologic impairment (%)	17.9 (1.1)	5.1 (0.3)	0.203
AMI (%)	10.8 (0.6)	35.9 (2.1)	0.222
CRRT (%)	19.8 (1.2)	41.8 (2.4)	0.374
IABP use (%)	209.0 (12.3)	430.9 (25.0)	0.012
Death (%)	39.4 (2.3)	9.8 (0.6)	0.144

AKI=acute kidney injury; AMI=acute myocardial infarction; BNP=brain natriuretic peptide; CABG=coronary artery bypass grafting; CRRT=continuous renal replacement therapy; EF=ejection fraction; IABP=intra-aortic balloon pump; ICU=intensive care unit; IPTW=inverse probability of treatment weighting; IQR=interquartile range; LCOS=low cardiac output syndrome; LVEDD=left ventricular end-diastolic diameter; LVESD=left ventricular end-systolic diameter; MR=mitral regurgitation; MVP=mitral valve plasty; OPCABG=off-pump coronary artery bypass grafting; SD=standard deviation; TNI=troponin I; TR=tricuspid regurgitation

### Follow-up Data

**Fig. 2:**
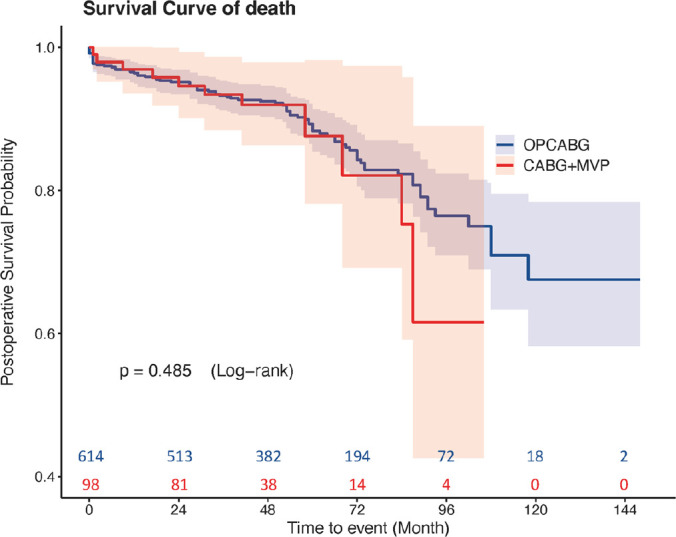
Unadjusted cumulative survival for the coronary artery bypass grafting (CABG) plus mitral valve plasty (MVP) and off-pump coronary artery bypass grafting (OPCABG) groups.

**Fig. 3:**
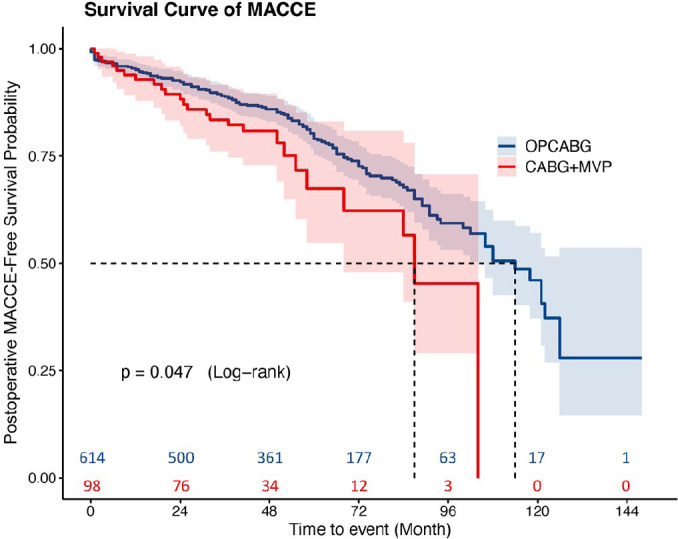
Unadjusted major adverse cardiac and cerebrovascular event (MACCE) rates for the coronary artery bypass grafting (CABG) plus mitral valve plasty (MVP) and off-pump coronary artery bypass grafting (OPCABG) groups.

**Fig. 4:**
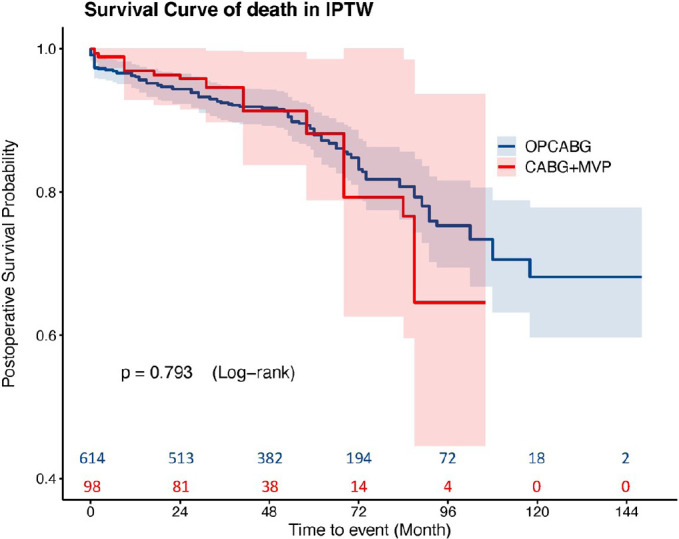
Cumulative survival of the coronary artery bypass grafting (CABG) plus mitral valve plasty (MVP) and off-pump coronary artery bypass grafting (OPCABG) groups after inverse probability of treatment weighting (IPTW).

**Fig. 5:**
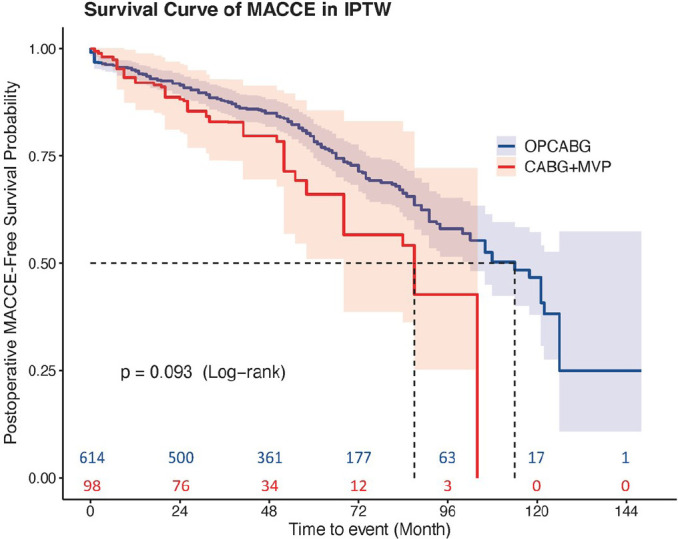
Major adverse cardiac and cerebrovascular event (MACCE) rates of the coronary artery bypass grafting (CABG) plus mitral valve plasty (MVP) and off-pump coronary artery bypass grafting (OPCABG) groups after inverse probability of treatment weighting (IPTW).

A total of 712 patients (86.6%) were followed up, and 110 patients (13.4%) were lost to follow-up. The median follow-up time was 58 months (range 12-148 months). There was no statistical difference in cumulative survival between the two groups (*P*=0.485) (Figure 2). MACCE events were significantly higher in the CABG+MVP group than in the OPCABG group (*P*=0.047) (Figure 3). There were no significant differences in the cumulative survival rate and the incidence of MACCE events between the two groups (*P*>0.05) after IPTW (Figures 4 and 5).

## DISCUSSION

Whether MVP should be performed with CABG in CHD patients with moderate IMR remains controversial^[3,4]^. Correct management of CHD with moderate IMR is of great significance to the prognosis of patients. This study showed no significant difference in earlyand long-term survival and MACCE events between the OPCABG and CABG+MVP groups. In a 20-year retrospective study, isolated CABG was shown to be associated with maximum survival in IMR patients^[6]^. A meta-analysis showed that CABG+MVP cannot decrease long-term mortality and increase aortic cross-clamping and cardiopulmonary bypass times^[7]^. Studies have revealed that although CABG+MVP reduces postoperative MR and improves early symptoms, it does not improve the long-term living status of the patients, nor does it validate the long-term survival benefit of combined surgery^[8,9]^.

At the same time, the largest randomized controlled study (Cardiothoracic Surgical Trials Network [CTSN] trial) showed that CABG+MVP does not improve postoperative survival, reduces the incidence of overall adverse events and hospital readmission, and it is associated with more early neurological events and supraventricular arrhythmias^[10,11]^. Our study showed that isolated OPCABG did not increase the all-cause mortality and the incidence of MACCE events in these patients. In addition, the operation time, postoperative LCOS, and IABP utilization rate were lower in the isolated OPCABG group (*P*<0.05). Previous studies reveal that most CABG groups in cardiac surgery centers used on-pump CABG for these patients^[10-14]^. In this study, OPCABG was used in all CABG groups. OPCABG avoids releasing inflammatory factors and activation of the complement and coagulation systems caused by cardiopulmonary bypass. Furthermore, OPCABG reduces surgical bleeding and blood transfusion, and avoids the nervous system complications caused by microthrombus and micro gas embolus, as well as the injury of perfusion lung and other organs^[15-18]^. At the same time, CABG+MVP adds complexity to the procedure due to the addition of MVP. The intraoperative myocardial blood supply was blocked, causing myocardial ischemia-reperfusion injury. For patients at high risk, especially those with advanced age, significant ascending aorta calcification, severe pulmonary disease, or high risk of cardiopulmonary bypass, OPCABG may be preferably performed by experienced surgeons to reduce the perioperative risk^[5,19]^.

Although this study discussed the comparison of two surgical methods, the final consideration should be what kind of patients are more suitable for which surgical method to achieve individualized accurate diagnosis and treatment. Several studies have shown that greater ejection fraction, greater posterior-inferior volume ratio, early operation timing after infarction, large mitral leaflet size, presence of viable myocardium, and absence of dyssynchrony between papillary muscles predict moderate improvement in IMR after isolated CABG^[8^.^20,21]^. CABG+MVP can reduce postoperative MR significantly in several previous studies, and we have the same result. However, the CTSN trial showed that the insufficiency of left ventricular reverse remodeling was not only due to residual MR, but also related to poor improvement of left ventricular wall motion after revascularization^[10,11]^. Some researchers have reported the value of myocardial viability in predicting left ventricular reverse remodeling^[22,23]^. Our previous study revealed that preoperative MI with abnormal anterior wall motion, more infarcted myocardium, and the number of MI segments connected to papillary muscle ≥ 2 may be risk factors for IMR recurrence after isolated CABG^[24]^. For patients with severe ischemia and large-scale MI, MR is less likely to improve after revascularization; more active combined surgical strategies can be adopted, but the risk of surgery should be balanced. Thus, OPCABG is feasible when considering the risk of combined surgery. The likely challenges for such patients are achieving individualized precision treatment, balancing the advantages and disadvantages of the two surgical methods, and maximizing the benefits to patients.

### Limitations

This study has the following limitations: firstly, this was a single-centre retrospective observational study. Therefore, prospective, multi-centre, and large-sample studies are needed to validate our results. Secondly, all patients included in this retrospective study were not randomly divided into the OPCABG group and the CABG+MVP group. Even if the IPTW was adopted, the selection bias between the two groups could not be eliminated. Thirdly, the present study did not continuously evaluate MR after surgery, mainly due to the large follow-up time span and wide geographical distribution of patients, resulting in more lost echocardiography. Finally, this study did not evaluate the myocardial viability of these patients before and after surgery. In addition, the study did not further explore the individualized treatment of surgical selection for these patients.

## CONCLUSION

Compared with isolated OPCABG, CABG+MVP did not reduce all-cause mortality or MACCE events in CHD patients with moderate IMR and extended operative time. In addition, the incidence of postoperative LCOS and IABP utilization rate was higher in the CABG+MVP group. Therefore, isolated OPCABG is an alternative surgical approach to treat CHD complicated with IMR in experienced cardiac surgery centers.
